# The headache registry of the German Migraine and Headache Society (DMKG): baseline data of the first 1,351 patients

**DOI:** 10.1186/s10194-022-01447-3

**Published:** 2022-07-01

**Authors:** Ruth Ruscheweyh, Theresa Klonowki, Gudrun Goßrau, Torsten Kraya, Charly Gaul, Andreas Straube, Tim Patrick Jürgens, Jörg Scheidt, Stefanie Förderreuther

**Affiliations:** 1 Department of Neurology, Ludwig Maximilians University Munich, Munich, Germany; 2 German Migraine and Headache Society, Frankfurt, Germany; 3 Department of Psychosomatic Medicine and Psychotherapy, Technical University of Munich, Munich, Germany; 4 Headache Outpatient Clinic, Pain Center, University Hospital and Faculty of Medicine Carl Gustav Carus, TU Dresden, Dresden, Germany; 5 Department of Neurology, Hospital Sankt Georg Leipzig gGmbH, Leipzig, Germany; 6 Headache Center Frankfurt, Frankfurt, Germany; 7 Department of Neurology, Headache Center North-East, University Medical Center Rostock, Rostock, Germany; 8 Department of Neurology, KMG Klinikum Güstrow, Güstrow, Germany; 9 Institute for Information Systems, University of Applied Sciences Hof, Hof, Germany

**Keywords:** Registry, Headache, Migraine, Germany, Acute headache Treatment, Preventive headache Treatment

## Abstract

**Background:**

Although good treatment options exist for many headache disorders, not all patients benefit and disability continues to be large. To design strategies for improving headache care, real-world data observing standard care is necessary. Therefore, the German Migraine and Headache Society (DMKG) has established the DMKG Headache Registry. Here we present methods and baseline data.

**Methods:**

Accredited German headache centers (clinic-based or private practice) can offer participation to their patients. Patients provide headache history, current headache load (including a mobile headache diary), medication and comorbidities and answer validated questionnaires, prior to their physician appointment. Physicians use these data as the base of their history taking, and add, change or confirm some central information. Before the next visit, patients are asked to update their data. Patients will continuously be included over the next years.

**Results:**

The present analysis is based on the first 1,351 patients (1110 females, 39.6 ± 12.9 years) with a completed first visit. Most participants had a migraine diagnosis. Participants had 14.4 ± 8.5 headache days and 7.7 ± 6.1 acute medication days per month and 63.9% had a migraine disability assessment (MIDAS) grade 4 (severe disability). 93.6% used at least one acute headache medication, most frequently a triptan (60.0%) or non-opioid analgesic (58.3%). 45.0% used at least one headache preventive medication, most frequently an antidepressant (11.4%, mostly amitriptyline 8.4%) or a CGRP(receptor) antibody (9.8%). Most common causes for discontinuation of preventive medication were lack of effect (54.2%) and side effects (43.3%).

**Conclusion:**

The DMKG Headache Registry allows to continuously monitor headache care at German headache centers in both a cross-sectional and a longitudinal approach.

**Trial registration:**

The DMKG Headache Registry is registered with the German Clinical Trials Register (DRKS 00021081).

## Background

Headache and migraine are among the diseases that cause most disability in Western countries [1]. New therapeutics for migraine have expanded our treatment options [2–4] but not all patients benefit from these treatments. Possible reasons are that patients do not seek medical help, are not offered adequate treatments, do not take them as advised, or for a too short time or at too low doses, have exaggerated expectations, or discontinue because of insufficient effect, poor tolerability or other causes. Some data on these issues are available both internationally and in Germany. E.g., the Eurolight study showed insufficient treatment with triptans and preventive therapy in several European countries, including Germany [5]. An international study on 1165 migraine patients showed that the most common reasons for discontinuation of migraine preventive therapy were insufficient effect and poor tolerability [6]. A US claims database analysis showed that persistence to oral migraine preventive medication in chronic migraine is on average 25% and 14% at 6 and 12 months, respectively, and even lower after switching to another preventive medication [7].

Nonetheless, to pinpoint the reasons for insufficient headache care, we need more detailed and specific data, also including the patient perspective. This has prompted the German Migraine and Headache Society (German: Deutsche Migräne- und Kopfschmerzgesellschaft, DMKG) to establish a Headache Registry. The DMKG Headache Registry systematically collects data from patients suffering from primary and secondary headaches according to the ICHD-3 classification [8], treated at multiple headache centers with DMKG-accredited physicians throughout Germany. Data collection includes retro- and prospective participant- and physician-entered data on headache history, features and treatment, concomitant disorders and sociodemographic data, questionnaires and headache diaries, both cross-sectionally and longitudinally. This will allow observing the quality of headache care in Germany and identifying deficits in a standardized, scientific way. This kind of data has the potential to significantly advance headache research, especially as it puts a focus on the patient perspective and patient-reported outcomes while at the same time validating the data for scientific purposes by including standardized entries from the treating physicians.

An additional aim of the DMKG Headache Registry is to support participants in maintaining a structured and complete headache documentation, both using the mobile headache diary application (DMKG-App) and the recurrent documentation in the web portal.

In the present manuscript, we describe the methods used for data collection and report baseline data for the first 1,351 patients.

## Methods

The DMKG Headache Registry was started in June 2020 and will continue recruiting for the next years. It collects data on sociodemographics, headache and concomitant disorders from headache patients of participating centers, both cross-sectionally and longitudinally (before and at every visit at the center and continuously via the headache diary). Patients enter data shortly before their first and follow-up physician appointments and provide specific consent to make them available to their treating physician. Physicians use entered data as the base for their history taking and add, change and confirm some central information to validate data for scientific use.

The DMKG Headache Registry research protocol was developed by the German Migraine and Headache Society taking into account the recommendations of the European Medicines Agency [9] and the Agency for Healthcare Research and Quality [10]. Its overarching scientific aim is to observe clinical care for headache patients in Germany and identify strengths and deficits. It is conducted in accordance with the Declaration of Helsinki and has been approved by the ethics committee of the Ludwig-Maximilians-University Munich (leading ethics committee, 20–004), and by the responsible ethics committee of every participating headache center. It is conducted according to European and German Data Protection laws and registered with the German Clinical Trials Register (DRKS 00,021,081). It is funded by the German Migraine and Headache Society and unrestricted grants from companies with an interest in headache research (at the time of publication, these are Eli Lilly, Novartis, Teva and Lundbeck). Supporting companies were given the opportunity to comment on the research protocol.

Researchers can apply to the German Migraine and Headache Society for project-specific data analyses. Applications must contain a detailed description of the planned project including the statistical analysis plan and will be reviewed by the Headache Registry’s Scientific Steering Committee. More information on the DMKG Headache Registry is available from kopfschmerzregister@dmkg.de.

### Headache centers and physicians

German headache centers (clinic-based or private practices) can apply for participation in the DMKG Headache Registry if at least one physician has obtained the German Headache and Facial Pain Certificate, issued by the German Migraine and Headache society on the basis of headache training credits, treatment reports and a visiting day in one of the main German headache centers. It has to be renewed every 5 years.

The registry started recruitment with a pilot phase at a few centers in the second half of 2020 and has continuously expanded since. Data in the present manuscript stem from a total of 16 centers, including private practices (Dr. Malzacher, Reutlingen; Dr. Menekes, Stuttgart; Dr. Kukowski, Göttingen/Hildesheim; Dr. Peikert, Bremen; Dr. Lewis, Stuttgart; Dr. Erbacher, Straubing; Dr. Friedrich, Ravensburg; Dr. Ermeling-Heuser, Bonn; Dr. Rambold, Mühldorf) and clinic-based headache centers (at the Departments of Neurology of the following clinics: Dr. Ruscheweyh, Ludwig-Maximilians University Munich (main facility); Dr. Förderreuther, Ludwig-Maximilians University Munich (downtown facility); Dr. Marziniak, kbo Isar-Amper-Klinikum, München-Ost; Dr. Goßrau, University Hospital and Faculty of Medicine Carl Gustav Carus, TU Dresden; Dr. Rimmele, University Medicine Rostock and Dr. Gaul, Migraine and Headache Clinic Königstein). Some clinic-based headache centers accept referrals only from neurologists, but in general referral sources of these centers are mixed, including self-referrals, primary care and specialists.

### Patients

Adult headache patients being treated or entering treatment at one of the participating headache centers are eligible for participation. At the time they make an appointment, they are invited to register with the Headache Registry. After informed consent, they are asked to fill a set of standardized questions and several validated questionnaires (see below) in the Headache Registry’s web portal prior to their appointment. In addition, they are encouraged to use the Headache Registry’s mobile application (DMKG-App) as headache diary.

Before each follow-up appointment, patients are asked to update their entries in the web portal. Follow-up data are not part of the present analysis.

Patients can participate independent from their type of health insurance (statutory, accounting for ~ 90% of the German population, or private). German health insurance covers for headache treatment, including drugs provided they are approved for treatment of the respective headache disorder. Some drugs (OnabotulinumtoxinA, CGRP(R) antibodies) are covered only for refractory and/or chronic migraine.

Patient engagement is kept high by making the entered information directly available to their treating physicians and by providing summaries (including graphical presentations) of headache diary and web portal data for download for the patients’ personal documentation.

### Collected data

**Fig. 1:**
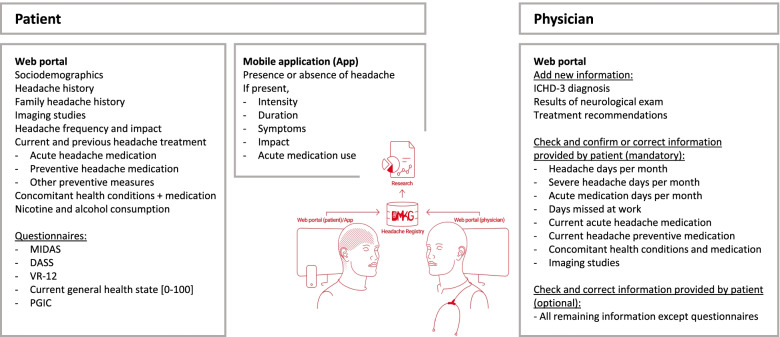
Overview of data capture within the DMKG Headache Registry. Data are entered by the patient before the first appointment and updated before every follow-up appointment. During or after the visit, the physician adds, corrects or confirms some central data and closes the visit. Assessment of headache treatment from the patient includes reasons for discontinuation for stopped treatments and rating of efficacy, tolerability, satisfaction with treatment and indication of side effects according to the GASE scale. PGIC is assessed only starting from the second visit. MIDAS, migraine disability assessment score; DASS, depression anxiety stress scales; VR-12, veterans RAND 12-item health survey; PGIC, patient global impression of change

During data entry, automated validation checks are constantly run and participants are asked to confirm and/or correct entries with implausible values. As the computerized data acquisition requires complete data entry, there is no missing data from the questionnaires or from the central headache and sociodemographic information. Figure 1 provides an overview of collected data.

#### Information about headache and concomitant disorders

At their first presentation within the Headache Registry, participants provide ample information regarding their headaches (e.g. start of headaches, number of headache days and severe headache days per month, number of medication days per month, number of days not being able to work, headache intensity, current and previous acute and preventive headache medication) and their non-headache health conditions and concomitant medication.

#### Questionnaires

Validated self-report questionnaires were selected on several grounds: (1) briefness, (2) license-free availability and (3) assessment of key headache-related factors (disability, depression, anxiety, stress, quality of life and current general state of health) using instruments broadly used in headache and pain research.

##### Migraine disability assessment scale (MIDAS) [11]

The MIDAS uses five items to measure headache-related disability within the last three months in the areas (a) work/school, (b) household and (c) family/social/leisure activities. It assesses number of days with complete disability (3 items), and with productiveness reduced by half or more (2 items). The MIDAS score (item sum) ranges from 0 to 279. Four grades of disability have been defined: grade 1 (MIDAS score 0–5) little or no disability; grade 2 (6–10) mild disability; grade 3 (11–20) moderate disability; grade 4 (≥ 21) severe disability.

##### Depression, Anxiety and Stress Scales, short form (DASS-21) [12, 13]

The DASS-21 measure depression, anxiety and stress levels within the last week using three 7-item subscales. Each item is scored on a 4-point scale ranging from 0 (did not at all apply to me) to 3 (applied to me very much or most of the time). Each subscale can reach a score between 0 and 21. Cut-offs for clinically significant values have been defined at ≥ 11 (depression and stress) and ≥ 7 (anxiety) [12].

##### Veterans RAND 12-item Health Survey (VR-12) [14, 15]

The VR-12 is a public domain questionnaire of health-related quality of life, similar to the extensively used Short Form-12 Health Survey (SF-12). 12 items are rated on Likert scales, and raw scores are transformed to scales from 0 to 100 using a dedicated algorithm, normalized to a healthy population with a mean of 50 and a standard deviation of 10. Two summary scores are obtained: the mental component score (MCS) evaluating general mental well-being and absence of psychological distress and the physical component score (PCS) assessing physical functioning. Larger scores indicate better quality of life.

In addition, current general state of health is assessed on a visual analogue scale (VAS, 0–100, 0 = worst, 100 = best).

At follow-up visits, the Patient Global Impression of Change (PGIC) on a 7 point Likert scale is also collected (not applicable for the present analysis).

#### Headache diary

Use of the Headache Registry’s mobile application (DMKG-App) as headache diary is encouraged, but not mandatory. Participants receive a daily reminder. The value of the daily entry can be ‘headache’, ‘no headache’ or ‘missing’. In the case of headache, further information is acquired: headache severity (on the NRS 0–10), headache duration (hours), headache features and concomitant symptoms, impact on daily activities and medication intake. The app allows entries only for the current day and up to 14 days in the past to avoid recall bias. Migraine days are distinguished from non-migraine days based on ICHD-3 criteria [8].

#### Data provided by physicians

At the physician appointment, entered data are used as the base for history taking. Treating physicians provide the ICHD-3 diagnosis [8] and confirm or correct some of the central patient’s entries (e.g. headache, severe headache, and medication days per month, current acute and preventive medications). After that, they close the visit. Physicians receive an appropriate financial compensation for their time (per closed visit).

### Analysis of baseline data

For the present baseline analysis, all patients who had a closed first physician visit within the Headache Registry before 03 March 2022 were included. Only data from the first visit were analysed.

Analysis was performed with R (version 4.0.5) and with SPSS (version 27). Descriptive statistics include mean ± standard deviation, median and interquartile range, and numbers and percentages as appropriate.

## Results

The present baseline analysis includes 1,351 patients, recruited at 16 centers. 10 of the centers were private practices (*n* = 720 patients) and 6 were headache centers at the university or other hospitals (*n* = 631).

**Fig. 2:**
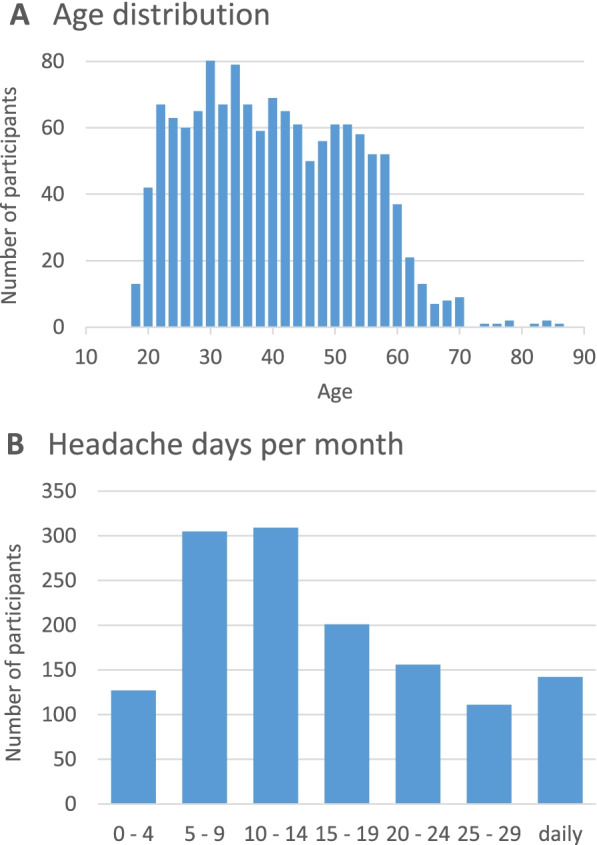
Distribution of age and headache days per month (*n* = 1,351)

See Table 1 for participants’ characteristics. Mean age was 39.6 ± 12.9 years (Fig. 2) and 1110 (82.2%) were female. Regarding school education, most participants had a university entrance diploma (usually corresponding to 12 or 13 school years). Regarding further education, most had a formal professional training or a university diploma. Nearly half of the participants worked full-time, but 22.8% did not work.

**Table 1:** Participant characteristics (*n* = 1351)

Age [years]		39.6 ± 12.9
Sex	Female	1110 (82.1%)
	Male	239 (17.7%)
	Diverse	2 (0.2%)
School education	No degree	4 (0.3%)
	Secondary education certificate (9 years)	106 (7.8%)
	Secondary education certificate (10 years)	414 (30.6%)
	Technical college entrance diploma (11–12 years)	167 (12.4%)
	University entrance diploma (12–13 years)	660 (48.9%)
Professional training	None	128 (9.5%)
	Formal professional training	653 (48.3%)
	University degree	494 (36.7%)
	Other	76 (5.3%)
Employment	Not working	308 (22.8%)
	Part-time	412 (30.5%)
	Full-time	631 (46.7%)

At the time of analysis, patients had completed between 1 and 8 visits at their headache center, amounting to a total of 2430 visits. Here, we analysed only data from the first visit.

75.9% of the patients used the Headache Registry’s mobile application as headache diary. In total, 70,616 headache days (of which 38,540 migraine days) and 98,529 headache free days had been recorded from these participants at the time of analysis.

Headache diagnoses are listed in Table 2. Most frequent diagnoses were migraine without aura (51.2%), chronic migraine (27.0%) and migraine with aura (26.9%), followed by medication overuse headache (MOH, 4.7%), tension-type headache (7.7%), new daily persistent headache (1.6%) and cluster headache (1.0%). As expected in the present setting, there was a number of less frequent headache diagnoses, including hemiplegic migraine, hemicrania continua, paroxysmal hemicrania and hypnic headache (see Table 2 legend for a full list).

**Table 2:** Headache diagnoses

	ICHD-3 diagnosis	Number of patients (%)
Migraine without aura	1.1	692 (51.2%)
Chronic migraine	1.3	365 (27.0%)
Migraine with aura	1.2	363 (26.9%)
Medication overuse headache	8.2	64 (4.7%)
Chronic tension-type headache	2.3	54 (4.0%)
Episodic tension-type headache	2.1/2.2	50 (3.7%)
(Probable) new daily persistent headache	4.10	22 (1.6%)
Cluster headache	3.1	14 (1.0%)

Diagnoses were not mutually exclusive, some (*n* = 309) participants received more than 1 (up to 4) diagnoses. Only diagnoses assigned to 10 or more participants are included in the Table. Migraine with aura also included: Typical aura without headache (*n* = 8), hemiplegic migraine (3) and migraine with brainstem aura (3). Other diagnoses under 10 occurrences included: Probable migraine without aura/with aura (*n* = 6/4), probable tension-type headache chronic/episodic (8/2), status migrainosus (8), (probable) hemicrania continua (6), persistent idiopathic facial pain (4), (probable) vestibular migraine (4), trigeminal neuralgia (3), (probable) primary stabbing headache (3), headache attributed to temporomandibular disorder (2), (probable) primary exercise headache (2), visual snow (2), painful trigeminal neuropathy (2), paroxysmal hemicrania (1), hypnic headache (1), persistent headache attributed to past non-traumatic intracranial haemorrhage (1),probable occipital neuralgia (1), probable cervicogenic headache (1), probable retinal migraine (1), probable nummular headache (1)

Headache characteristics and results of the questionnaires are shown in Table 3. Participants were on average severely affected, with an average number of headache days per month of 14.4 ± 8.5 (Fig. 2) and 63.9% falling in MIDAS grade 4 (severe disability). 17.2% and 18.9% had DASS depression and anxiety scores elevated above the cut-off, respectively.

**Table 3:** Headache characteristics and results of questionnaires

Headache days per month^a^	14.4 ± 8.5
- 0–3 days per month	87 (6.4%)
- 4–7 days per month	214 (17.8%)
- 8–14 days per month	413 (30.6%)
- 15 and more days per month	610 (45.2%)
Severe headache days per month^a^	7.6 ± 5.9
Acute medication days per month^a^	7.7 ± 6.1
Days lost at work per month^a^	3.1 ± 6.2
Days lost in household work per month^a^	5.9 ± 6.0
Headache intensity [0–10]	6.4 ± 1.8
Headache duration [years]	18.4 ± 14.0
MIDAS score [0–279]	47.4 ± 50.2
- Grade 1 (0–5)	136 (10.1%)
- Grade 2 (6–10)	100 (7.4%)
- Grade 3 (11–20)	252 (18.7%)
- Grade 4 (> 20)	863 (63.9%)
DASS depression score [0–21]	5.6 ± 5.0
- above cutoff (≥ 11)	232 (17.2%)
DASS anxiety score [0–21]	3.6 ± 3.7
- above cutoff (≥ 7)	256 (18.9%)
DASS stress score [0–21]	7.8 ± 5.0
- above cutoff (≥ 11)	391 (28.9%)
VR-12 Mental component score (MCS)	41.8 ± 11.2
VR-12 Physical component score (PCS)	40.9 ± 9.1
Current general health state [0–100]	53.6 ± 22.8

^a^average of past 3 months

*MIDAS* Migraine disability assessment score, *DASS* Depression anxiety stress scales, *VR-12* Veterans RAND 12-item health survey

The most frequent classes of concomitant health conditions were allergies (*n* = 577, 42.7%), other pain disorders (*n* = 504, 37.3%) and psychiatric disorders (*n* = 504, 37.3%) (see Table 4 for a list of the single health conditions).

**Table 4:** Concomitant health conditions

	Number of patients (%)
Allergic rhinitis	324 (24.0%)
Other health conditions	306 (22.7%)
Allergies, other	293 (21.7%)
Temporomandibular dysfunction	278 (20.7%)
Sleep disturbances	277 (20.5%)
Chronic back pain (cervical)	229 (17.0%)
Hypothyroidism	224 (16.6%)
Depression, active	184 (13.6%)
Allergic reactions to medication	179 (13.2%)
Chronic back pain (lumbar)	178 (13.2%)
Anxiety disorder/panic disorder	153 (11.3%)
Asthma	129 (9.6%)
High blood pressure	111 (8.2%)
Depression, remitted	103 (7.6%)

Only health conditions indicated by 100 or more participants are included in the Table. Non-headache health conditions were selected from a list, with the option to select “other” followed by a free-text field. Please note that some participants had several health conditions from the same class (e.g. allergies), so that the sum (e.g. of the different allergic conditions) exceeds the frequency given in the text for the class

**Fig. 3:**
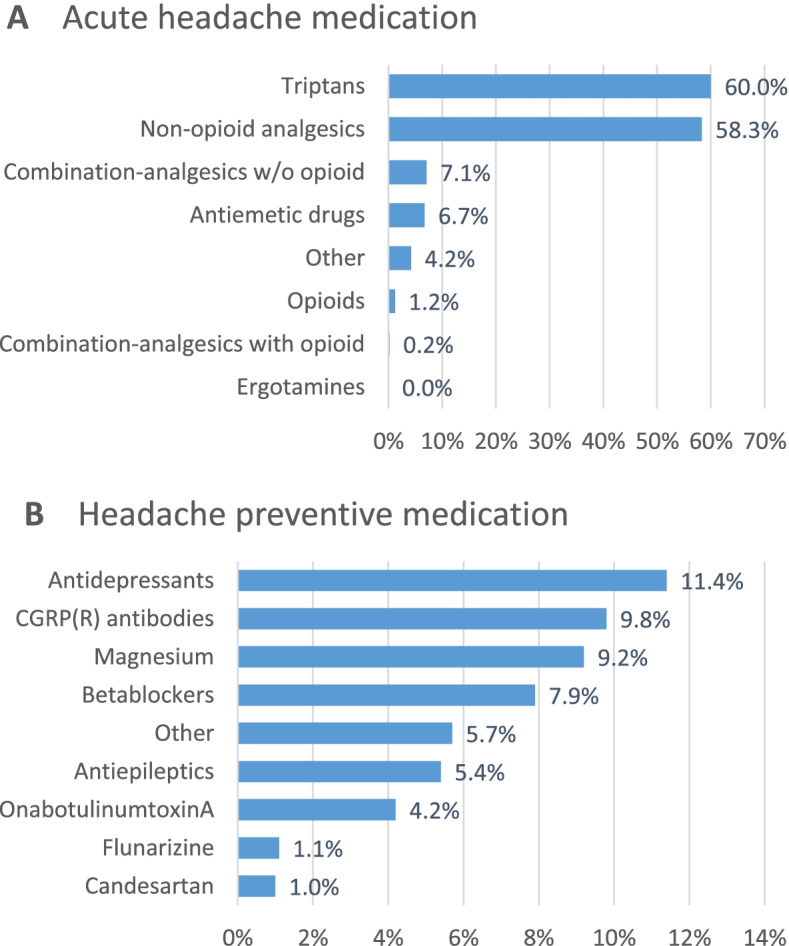
Acute headache medication and headache preventive medication. 6.4% of patients did not use acute headache medication and 55% did not use headache preventive medication. Antidepressants: tricyclics and serotonin-noradrenaline reuptake inhibitors (SNRI); CGRP and CGRP receptor antibodies (CGRP(R) antibodies): erenumab, fremanezumab, galcanezumab; magnesium: including combinations with vitamin B2 and coenzyme Q10; antiepileptics: topiramate and valproate. w/o, without

Headache medication is shown in Fig. 3. Almost all patients (93.6%) used acute headache medication (mostly 1 or 2 but up to 9 different substances). Triptans (60.0%) and non-opoid analgesics (58.3%) were by far the most frequently used categories. 45.0% of the patients used one or more (up to 5) preventive headache medications. The most frequently used classes were antidepressants (11.4%), CGRP(R) antibodies (9.8%) and magnesium (alone and in combination with vitamin B2 and coenzyme Q10, 9.2%), see Table 5 for details.

**Table 5:** Preventive headache medication

Group	Substance	Number of patients (%)
Antidepressants	Amitriptyline	113 (8.4%)
	Opipramol	10 (0.7%)
	Amitriptylinoxide	5 (0.4%)
	Doxepin	3 (0.2%)
	Duloxetine	11 (0.8%)
	Venlafaxine	14 (1.0%)
Antiepileptics	Topiramat	71 (5.3%)
	Valproate	2 (0.1%)
Betablockers	Bisoprolol	16 (1.2%)
	Metoprolol	83 (6.1%)
	Propranolol	8 (0.6%)
Botulinum neurotoxin	OnabotulinumtoxinA	56 (4.2%)
CGRP(R) antibodies	Erenumab	73 (5.4%)
	Fremanezumab	31 (2.3%)
	Galcanezumab	31 (2.3%)
Other substances	Flunarizine	15 (1.1%)
	Candesartan	13 (1.0%)
	Lisinopril	0 (0.0%)
	Acetylsalicylic acid	2 (0.1%)
	Magnesium	98 (7.3%)
	Magnesium + vitamin B2 + coenzyme Q10	26 (1.9%)
	Other	75 (5.6%)

36.9% of the patients reported previous attempts with preventive headache medication (between 1 and 13 attempts). The most frequent reasons for discontinuation were: no effect (54.2%), poor tolerability (43.3%), partial but insufficient effect (15.7%), good effect but poor tolerability (2.4%) and other (9.4%) (multiple selections possible).

## Discussion

The present manuscript describes the methods and the baseline data of the first 1,351 patients of the DMKG Headache Registry.

What can the DMKG Headache Registry contribute to headache research?

Registries are important research tools because they capture real-world treatment as performed during standard clinical care, encompassing a broad range of patients, with a minimum of inclusion and exclusion criteria. Depending on their design, they can provide important information on patient histories and trajectories, treatment attempts and their success, and the patients’ perspective.

The DMKG Headache Registry captures previous headache history and both cross-sectional and longitudinal data. It therefore can provide information about previous and current treatment, but also prospectively follow treatments initiated during the observation period, using headache diary data to evaluate treatment success. It characterizes headache patients with respect to sociodemographics, comorbidities, and healthcare resource utilization. It can provide a picture of current standard clinical care in Germany, allowing identification of deficits that can subsequently be addressed. In addition, Headache Registry data could help determining predictors of successful treatment although confounding by individual selection of treatments must be considered.

The Headache Registry captures data provided by both the patient and the treating physician. Assessing the patient’s perspective can give important information on why headache treatment works for some patients but not for others. It can also help identifying treatment barriers and therefore contribute to designing more effective treatment strategies. The DMKG Headache Registry uses patient-reported outcome measures (PROMs) in the form of validated questionnaires to assess disability, quality of life, depression, anxiety and stress. In addition, patients rate effect and tolerability of treatments, and give reasons for treatment discontinuation.

By including patients with all types of headache, the DMKG Headache Registry is able to identify patients with rare headache disorders from all over Germany. Over time, groups of meaningful size will accumulate, allowing to analyse course of disease and responses to treatment of rare headache disorders or even to invite these patients for participation in dedicated studies. Indeed, the Headache Registry has already collected some data from rare headache disorders like hemiplegic migraine, hemicrania continua, paroxysmal hemicrania and hypnic headache. It must be noted that as development of a module better suited for patients with short-lasting headache attacks such as cluster headache has been announced, these patients may currently be underrepresented in the Registry.

If they do not focus on a specific diagnosis or treatment, registries need to include large numbers of patients so than meaningful subgroups can be formed. Therefore, a compromise between the extent, completeness, quality and internal and external validation of the data on the one hand and the ease of participation and data capturing for both patients and physicians on the other hand has to be found. In the DMKG Headache Registry, we keep data quality high by making the German Headache and Facial Pain Certificate a pre-requisite of physician participation. In addition, we demand physicians to provide key information (such as the diagnosis) and confirm or correct some of the central patient’s entries (e.g. headache days, severe headache days, and medication days per month, current acute and preventive medications). However, we refrain from asking physicians or study nurses from manually entering headache history and treatment information into a separate physician’s system, and we do not perform routine monitoring. At the patient’s side, the Registry provides easy-to-use surfaces in both the web portal and the DMKG-App with automated validation checks during data entry. Patients are motivated to keep their records in the Headache Registry, because the entered information is directly made available to their treating physicians and can serve as the base for their physician’s appointment.

### Other headache registries

The idea of collecting headache patients from multiple centres in a registry is not new, but digitalization has made this kind of data collection much easier. The American Registry for Migraine Research (ARMR) collects data since 2016 and published its methods and initial patient cohort in 2019 [16]. Several publications have followed [17–19], giving examples of what types of analysis can be performed with registry data. The ARMR in addition to history, treatment data and PROMs also collects routine imaging data and blood samples. There is a Chinese Headache Registry that started recruitment in 2021 (NCT04939922) with no published data up to now. A German registry of different neurological disorders including headache (NeuroTransData) has published some data in abstract form [20]. There is also a Children and Adolescent Migraine Registry from the US [21] and some more specific registries like the Korean Cluster Headache Registry [22] or the Spanish registry for the use of erenumab for migraine prevention [23]. The list is not complete and more will surely follow in the next years.

### Baseline data from the DMKG headache registry

The present analysis of the first 1,351 patients shows that, as expected, most patients had a migraine diagnosis. Consistent with the female preponderance of migraine, > 80% of the patients were female. The patients were on average severely affected, with 14.4 ± 8.5 headache days per month, and 63.9% falling into MIDAS grade IV (severe disability). For comparison, patients in the ARMR are even more severely affected, with an average number of 19.1 ± 9.2 headache days per month and 67.2% falling into MIDAS grade 4 [16]. Maybe in association, our patients were also somewhat younger (39.6 ± 12.9 years) than those of the ARMR (48.6 ± 13.9). It might be surprising that a diagnosis of MOH was made in only 4.7% of this severely affected sample. This is a phenomenon also encountered in the ARMR, and under-utilization of this diagnosis has been discussed [16]. In addition, trigemino-autonomic headaches may currently be underrepresented in the DMKG Headache Registry, as participating centers were informed that a special cluster headache module would be provided later. The proportion of chronic tension-type headache may also seem low. This might be related to the new chronic migraine criteria in IDCH-3 [8] compared to ICHD-2R [24], with many patients previously classified as having episodic migraine plus chronic tension-type headache now receiving a chronic migraine diagnosis. Moreover, with the exception of MOH, the initial focus of the DMKG Headache Registry was on primary headaches. A recent update will improve capture of secondary headache diagnoses.

Most common comorbidities were allergies, other pain disorders and psychiatric disorders. This is similar to previous reports [25, 26] and to ARMR data [16].

Acute headache medication is used by almost all patients, with triptans and non-opioid analgesics being the most frequent, again similar to the ARMR data. The relatively high frequency of triptan use (60.0%) probably reflects a severely affected patient population being referred to or treated at specialized headache centers.

The proportion of patients using a preventive headache medication however was much lower in the present sample (45.0%) compared to the ARMR (84.7%). This is probably due to the fact that many patients came to their first appointment at the specialized headache center when first participating in the Headache Registry. Longitudinal analysis will show if proportions of patients with preventive headache medication increase at follow-up visits. Nonetheless, about one third of the patients reported previous attempts with headache preventive medication, so that the relatively low proportions of betablockers, antiepileptics and onabotulinumtoxinA among the current preventive medications might in part be due to patients already having tried these medications. However, other reasons such as reluctance to prescribe or take certain drugs or limited knowledge about headache preventive medication likely also play a role [27, 28]. Magnesium is frequently used for migraine prevention in Germany, with a high patient preference because of its good tolerability, although the national guideline lists it only as a secondary option. The present and previous data show that patterns of preventive medication use are complex and may depend on multiple factors. We thus plan to conduct a more detailed analysis of previous and present preventive medication for a separate publication. Interestingly, the most frequent reasons for discontinuation of previous preventive medication were no effect (54.2%) and poor tolerability (43.3%). These results compare to data from a multinational survey reporting lack of efficacy in 37–48% of patients and side effects in 34–53% as the reason for termination [6]. It will be interesting to see how these data break down to different classes of preventive medication, including the new class of CGRP(receptor) antibodies.

The above comparison illustrates similarities and differences to the ARMR data. Other differences between the two registries include local health systems and treatment recommendations and maybe also patient characteristics such as attitudes towards treatment. This underlines the importance of having a headache registry also in Germany.

### Strength and limitations

An important strength of the DMKG Headache Registry is the focus on patient-reported data while at the same time validating the entries by accredited treating physicians. Another strength is the double aim of generating data for research but also supporting patients with their headache documentation and allow data sharing with the treating physician, which increases the patients’ engagement. Imaging data and blood samples are currently not collected, which precludes some types of analysis but makes participation easier for both physicians and patients. It must be noted that different from population surveys which are also a field of research of the German Migraine and Headache Society [29], registry data can never be representative for the expression of the disorder in the general population. First, persons with headache that do not see a physician are not captured at all. Second, the DMKG Headache Registry currently focuses on centers with a special interest in headache, which keeps data quality high, but results in a significant selection bias towards more severely affected patients. This problem is challenging, as mainly physicians with a special interest in headache are willing to participate in such a registry. However, we plan to implement a basic module adapted for headache treatment in primary care in the future. Last but not least, although electronic data capture has largely facilitated registry conduction, some patients may not want to or be able to use electronic devices for headache documentation. On the other hand, registry data is always observational. Resulting risks of bias include the lack of random assignment to therapies and the possible impact of treatment success on patients’ willingness to participate. In addition, data from a national registry cannot be directly extrapolated to other countries. Nonetheless, thanks to the International Classification of Headache Disorders [8] and international guidelines [2, 30] comparison between data from different countries is largely facilitated.

## Conclusions

The present report provides an overview of the methods and basic results of the first 1351 patients of the DMKG Headache Registry. The Registry will continuously recruit patients and additional centers to provide up-to-date, detailed, cross-sectional and longitudinal real-world evidence on headache care in Germany. Several expansions are possible and/or planned, such as addition of a dedicated cluster headache module, of a facial pain module and of a basic module that facilitates participation of general practitioners with an interest in headache.

## Data Availability

The datasets analysed during the current study are available from the German Migraine and Headache Society on reasonable request. Please note that access to data principally suitable for conducting additional analyses must be reviewed by the Headache Registry’s Scientific Steering Committee.

## Abbreviations

DMKG: Deutsche Migräne- und Kopfschmerzgesellschaft (German headache and migraine society)
MIDAS: Migraine disability assessment score
DASS: Depression anxiety stress scales
VR-12: Veterans RAND 12-item health survey
VAS: Visual analogue scale
CGRP: Calcitonin gene-related peptide
MCS: Mental component score (of the VR-12)
PCS: Physical component score (of the VR-12)
PGIC: Patient global impression of change
NRS: Numerical rating scale
ICHD-3: International classification of headache disorders, 3^rd^ edition
MOH: Medication overuse headache
ARMR: American Registry for Migraine Research

## References

[1] GBD (2016). Neurology Collaborators (2019) Global, regional, and national burden of neurological disorders, 1990–2016: a systematic analysis for the Global Burden of Disease Study 2016. Lancet Neurol.

[2] Sacco S, Bendtsen L, Ashina M (2019). European headache federation guideline on the use of monoclonal antibodies acting on the calcitonin gene related peptide or its receptor for migraine prevention. J Headache Pain.

[3] Diener H-C, Holle-Lee D, Nägel S (2019). Treatment of migraine attacks and prevention of migraine: Guidelines by the German Migraine and Headache Society and the German Society of Neurology. Clin Transl Neurosci.

[4] Ailani J, Burch RC, Robbins MS, Board of Directors of the American Headache Society (2021). The American headache society consensus statement: update on integrating new migraine treatments into clinical practice. Headache.

[5] Katsarava Z, Mania M, Lampl C (2018). Poor medical care for people with migraine in Europe - evidence from the Eurolight study. J Headache Pain.

[6] Blumenfeld AM, Bloudek LM, Becker WJ (2013). Patterns of use and reasons for discontinuation of prophylactic medications for episodic migraine and chronic migraine: results from the second international burden of migraine study (IBMS-II). Headache.

[7] Hepp Z, Dodick DW, Varon SF (2017). Persistence and switching patterns of oral migraine prophylactic medications among patients with chronic migraine: a retrospective claims analysis. Cephalalgia Int J Headache.

[8] Headache Classification Committee of the International Headache Society (IHS) (2018) The International Classification of Headache Disorders, 3rd edition. Cephalalgia Int J Headache 38:1–211. 10.1177/0333102417738202 10.1177/0333102417738202 29368949

[9] (2020) Guideline on registry-based studies – Draft. https://www.ema.europa.eu/en/guideline-registry-based-studies (2020, accessed 23 Mar 2022).

[10] Gliklich RE, Leavy MB, Dreyer NA (2020) Registries for Evaluating Patient Outcomes: A User’s Guide. 4th ed. (Prepared by L&M Policy Research, LLC, under Contract No. 290-2014-00004-C with partners OM1 and IQVIA) AHRQ Publication No. 19(20)-EHC020. Rockville: Agency for Healthcare Research and Quality. Posted final reports are located on the Effective Health Care Program search page. 10.23970/AHRQEPCREGISTRIES4

[11] Stewart WF, Lipton RB, Whyte J (1999). An international study to assess reliability of the Migraine Disability Assessment (MIDAS) score. Neurology.

[12] Nilges P, Essau C (2015). Depression, anxiety and stress scales: DASS–A screening procedure not only for pain patients. Schmerz Berl Ger.

[13] Lovibond SH, Lovibond PF (1996). Manual for the depression anxiety stress scales.

[14] Kazis LE, Miller DR, Skinner KM (2004). Patient-reported measures of health: the Veterans health study. J Ambulatory Care Manage.

[15] Hüppe M, Schneider K, Casser H-R (2022). Characteristic values and test statistical goodness of the Veterans RAND 12-Item Health Survey (VR-12) in patients with chronic pain : an evaluation based on the KEDOQ pain dataset. Schmerz Berl Ger.

[16] Schwedt TJ, Digre K, Tepper SJ (2020). The American registry for migraine research: research methods and baseline data for an initial patient cohort. Headache.

[17] Pearl TA, Dumkrieger G, Chong CD (2020). Sensory hypersensitivity symptoms in migraine with vs without aura: results from the American registry for migraine research. Headache.

[18] Pearl TA, Dumkrieger G, Chong CD (2020). Impact of depression and anxiety symptoms on patient-reported outcomes in patients with migraine: results from the American Registry for Migraine Research (ARMR). Headache.

[19] Trivedi M, Dumkrieger G, Chong CD (2021). Impact of abuse on migraine-related sensory hypersensitivity symptoms: Results from the American Registry for Migraine Research. Headache.

[20] Peikert A, Koerwer M, Tozzi V, et al (2020) First insights in real-world effectiveness of erenumab in chronic migraine patients with high burden of disease in Germany from the NTD headache and migraine registry (Abstract 1760). Neurology. 94(15 Supplement)

[21] Hornik CP, Gelfand AA, Szperka CL (2020). Development of a prospective real-world data clinical registry of children and adolescents with migraine. Headache.

[22] Cho S-J, Lee MJ, Kim B-K, et al (2019) Clinical features of chronic cluster headache based on the third edition of the International Classification of Headache Disorders: a prospective multicentre study. PloS One. 14:e0221155. 10.1371/journal.pone.0221155 10.1371/journal.pone.0221155 PMC6709915 31449536

[23] Belvís R, Irimia P, Pozo-Rosich P (2021). MAB-MIG: registry of the spanish neurological society of erenumab for migraine prevention. J Headache Pain.

[24] Silberstein SD, Olesen J, Bousser M-G, et al (2005) The International Classification of Headache Disorders, 2nd Edition (ICHD-II)--revision of criteria for 8.2 Medication-overuse headache. Cephalalgia Int J Headache. 25:460–465. 10.1111/j.1468-2982.2005.00878.x 10.1111/j.1468-2982.2005.00878.x 15910572

[25] Dresler T, Caratozzolo S, Guldolf K (2019). Understanding the nature of psychiatric comorbidity in migraine: a systematic review focused on interactions and treatment implications. J Headache Pain.

[26] Le H, Tfelt-Hansen P, Russell MB (2011). Co-morbidity of migraine with somatic disease in a large population-based study. Cephalalgia Int J Headache.

[27] Dekker F, Dieleman JP, Neven AK (2013). Preventive treatment for migraine in primary care, a population-based study in the Netherlands. Cephalalgia Int J Headache.

[28] Dekker F, Neven AK, Andriesse B (2012). Prophylactic treatment of migraine by GPs: a qualitative study. Br J Gen Pract J R Coll Gen Pract.

[29] Müller B, Dresler T, Gaul C (2020). Use of outpatient medical care by headache patients in Germany: a population-based cross-sectional study. J Headache Pain.

[30] Steiner TJ, Jensen R, Katsarava Z (2019). Aids to management of headache disorders in primary care (2nd edition): on behalf of the European Headache Federation and Lifting The Burden: the Global Campaign against Headache. J Headache Pain.

