# Correction: The headache registry of the German Migraine and Headache Society (DMKG): baseline data of the first 1,351 patients

**DOI:** 10.1186/s10194-022-01453-5

**Published:** 2022-07-15

**Authors:** Ruth Ruscheweyh, Theresa Klonowski, Gudrun Goßrau, Torsten Kraya, Charly Gaul, Andreas Straube, Tim Patrick Jürgens, Jörg Scheidt, Stefanie Förderreuther

**Affiliations:** 1 Department of Neurology, Ludwig Maximilians University Munich, Munich, Germany; 2 German Migraine and Headache Society, Frankfurt, Germany; 3 Department of Psychosomatic Medicine and Psychotherapy, Technical University of Munich, Munich, Germany; 4 Headache Outpatient Clinic, Pain Center, University Hospital and Faculty of Medicine Carl Gustav Carus, TU Dresden, Dresden, Germany; 5 Department of Neurology, Hospital Sankt Georg Leipzig gGmbH, Leipzig, Germany; 6 Headache Center Frankfurt, Frankfurt, Germany; 7 Department of Neurology, Headache Center North-East, University Medical Center Rostock, Rostock, Germany; 8 Department of Neurology, KMG Klinikum Güstrow, Güstrow, Germany; 9 Institute for Information Systems, University of Applied Sciences Hof, Hof, Germany

**Correction: J Headache Pain 23, 74 (2022)**

**https://doi.org/10.1186/s10194-022-01447-3**

Following publication of the original article [1], the authors identified an error in the author name of Theresa Klonowski.

The incorrect author name is: Theresa Klonowki.

The correct author name is: Theresa Klonowski.

The author group has been updated above and the original article [1] has been corrected.
